# Urban-rural differences in the prevalence and influencing factors of insomnia: a cross-sectional study in Foshan, China

**DOI:** 10.3389/fpsyt.2025.1580013

**Published:** 2025-06-27

**Authors:** Zhaosong Chu, Jianling Xie, Rui Liu, Liqin Ou, Zheng Lan, Jiaquan Liang, Guojun Xie

**Affiliations:** ^1^ Department of Clinical Psychology, The Third People’s Hospital of Foshan, Foshan, China; ^2^ Department of Public Health, The Third People’s Hospital of Foshan, Foshan, China; ^3^ Department of Psychiatry, The Xinshi Hospital of Gaoming District, Foshan, China

**Keywords:** insomnia, urban-rural differences, status survey, influencing factors, China

## Abstract

**Objective:**

Insomnia is a common sleep disorder that affects quality of life and varies significantly across populations. In China, the urban-rural divide is notable, but differences in insomnia prevalence and influencing factors between these groups remain unclear. Using Foshan as a case study, this research aims to identify these differences to develop targeted interventions for improving sleep quality.

**Methods:**

Data were obtained from the 2022 Foshan City Residents’ Mental Health Survey, which included 9,044 adult participants (4,546 urban and 4,498 rural residents). Insomnia was assessed using the Insomnia Severity Index, with participants scoring ≥ 8 classified as the insomnia group. Chi-square tests and logistic regression analyses were used to explore urban-rural differences in insomnia prevalence and influencing factors.

**Results:**

The prevalence of insomnia in Foshan was 18.37%. Urban residents exhibited a significantly higher insomnia rate (19.73%) compared to rural residents (16.99%). Logistic regression analysis indicated that among urban residents, being unmarried, widowed or divorced, engaging in professional technical work, chronic diseases, depression, and anxiety were risk factors for insomnia. Meanwhile, 6–12 years of education, regular dietary habits, and daily physical exercise were protective factors. For rural residents, female gender, high educational attainment, high monthly household income, chronic diseases, depression, and anxiety were risk factors, while regular diet and daily physical exercise emerged as protective factors.

**Conclusion:**

Insomnia prevalence is higher among urban residents in Foshan compared to rural residents, with distinct influencing factors for each group. Findings inform tailored interventions aimed at reducing insomnia disparities across urban and rural populations.

## 1 Introduction

Insomnia is defined as the subjective dissatisfaction with sleep duration or quality despite adequate opportunities and environments for sleep, manifesting primarily as difficulty initiating sleep, maintaining sleep, or early awakening, and leading to significant daytime functional impairment. It is estimated that around one-third of the world’s population experiences insomnia to varying degrees (1). A global systematic review showed that the prevalence of insomnia symptoms was even as high as 52.57% during COVID-19 (2). A cross-sectional study involving 512,891 Chinese adults aged 30–79 years found that 17% of adults reported insomnia symptoms (3). These data suggest that insomnia has become a major public health issue affecting the health of global populations.

Studies have found that insomnia not only impairs cognitive function and work efficiency but also increases the risk of workplace accidents (4, 5). Notably, chronic insomnia is strongly linked to elevated risks of numerous physical illnesses (such as cardiovascular diseases, stroke, chronic kidney disease) and psychological disorders (such as suicide, anxiety, depression, and Alzheimer’s disease) (6–9). Hence, investigating the underlying factors of insomnia is crucial for early prevention, precise diagnosis, and effective therapeutic interventions.

Previous studies have shown that demographic characteristics, health-related factors, and lifestyle factors significantly affect the occurrence of insomnia. More specifically, demographic factors including age, gender, education level, and income status are strongly correlated with the prevalence of insomnia. Salo et al. found that the prevalence of insomnia is significantly higher in older adults compared to middle-aged and young adults (34–45 years old) (10). Additionally, meta-analysis results show that women generally have a higher risk of insomnia compared to men (11). Studies on African and Latin American populations have found that the insomnia rate in low-education and low-income groups is significantly higher than in high-income groups (12, 13). In terms of health-related factors, chronic diseases (14), anxiety disorders (15), and depression (16) have all been shown to be closely associated with the occurrence of insomnia. With regard to lifestyle, harmful habits such as smoking (17), excessive alcohol consumption (18), and sedentary behavior (19) significantly raise the risk of insomnia, whereas consistent physical exercise is considered beneficial for sleep quality (20).

In China, the urban-rural dual structure has resulted in substantial differences between urban and rural populations regarding lifestyle, work environments, social support, and cultural perceptions. Such disparities may significantly influence the prevalence and determinants of insomnia, while also contributing to inequalities in insomnia management and access to healthcare resources across groups. Urban residents, for instance, may encounter elevated risks of insomnia driven by higher work stress and social competition, whereas rural populations may suffer due to inadequate social support and scarce healthcare resources. Such contextual differences underscore the intricate mental health challenges faced by urban and rural populations, emphasizing the need for tailored public health interventions.

Although previous studies have explored the prevalence of insomnia and its urban-rural disparities in China, many of these studies have primarily reported overall prevalence rates without addressing the underlying factors contributing to these differences (21, 22). Moreover, the results from these studies have shown significant heterogeneity. Some studies report a higher prevalence of insomnia among rural residents (22), while others indicate higher rates among urban populations (23, 24), or find no significant difference between the two groups (21, 25). This inconsistency may be attributed to a range of factors, including geographical diversity in study populations, variations in social, economic, and environmental contexts, and regional disparities in healthcare access. Additionally, the relatively small sample sizes in some studies may further contribute to the observed heterogeneity (21, 22).

Given the conflicting findings and the complex nature of urban-rural disparities in insomnia, there is a clear need for further research that delves deeper into the factors driving these differences. The present study, based on data from the 2022 Foshan City Residents’ Mental Health Survey, aims to explore the prevalence of insomnia among urban and rural adults and identify potential influencing factors for both groups. By examining these disparities, we aim to uncover actionable intervention points that could inform targeted public health strategies tailored to urban and rural populations. This evidence-based guidance will be critical for policymakers, helping to improve public health policy design and ultimately enhance mental health outcomes across different demographic groups.

## 2 Methods

### 2.1 Participants

This study utilized cross-sectional data from the 2022 Foshan City Residents’ Mental Health Survey, conducted from June to October 2022. A multi-stage stratified random sampling method was adopted to recruit adult permanent residents of Foshan as the research subjects. The inclusion criteria included: (1) age > 18 years; (2) having lived locally for over 6 months; (3) the ability to comprehend the questionnaire and actively participate in the survey. The exclusion criteria consisted of: (1) inability to complete the questionnaire due to cognitive impairment, mental disorders, or severe physical illness; (2) failure to contact the individual after at least 3 follow-ups.

### 2.2 Procedures and measurements

The sampling process consisted of three stages. In the first stage, the five districts of Foshan were stratified based on their financial status, and a proportional number of villages or residents’ committees were selected, ensuring representation across population sizes. A total of 89 villages or residents’ committees were chosen as primary sampling units. In the second stage, households in the selected areas were assessed, excluding empty, commercial, or invalid addresses. A systematic sampling method was then applied to select 105 households from each community, ensuring random selection based on door numbers. In the third stage, eligible adult residents within the selected households were registered, and one individual from each household was randomly chosen for the survey. Data collection was conducted by investigators who received uniform training. Participants were guided by investigators to scan the QR code of the “Foshan Mental Health Service Platform” to fill out the electronic questionnaire. For participants unable to use a mobile phone, data were collected through paper questionnaires or face-to-face interviews.

The questionnaire captured sociodemographic details (e.g., gender, age, educational attainment, marital status, occupation, and income level), lifestyle factors (e.g., smoking, alcohol consumption, tea drinking, napping, diet, and exercise frequency), and chronic disease history. Dietary habits encompass various dimensions, including the types of food consumed, portion sizes, cooking methods, and meal regularity. However, due to the complexity and diversity of dietary patterns among Chinese residents, it is challenging to assess all these factors comprehensively in large-scale epidemiological surveys. Therefore, in this study, we focused specifically on meal regularity. Dietary habits were categorized into two groups based on regularity: a “regular diet”, defined as eating meals (including breakfast, lunch, and dinner) at consistent times almost every day, and an “irregular diet”, defined as skipping any meals or having inconsistent meal timings. Physical exercise was defined as intentional activities such as walking or running, with a minimum duration of 10 minutes. Participants were categorized based on exercise frequency into four groups: “Hardly”, “1–2 times/week”, “3–5 times/week”, and “almost daily”.

The Chinese versions of the Insomnia Severity Index (ISI), Patient Health Questionnaire-9 (PHQ-9), and Generalized Anxiety Disorder-7 (GAD-7) were used to screen for insomnia, depression, and anxiety, respectively. The ISI consists of 7 self-reported items, each scored from 0 to 4, with a total score range of 0-28. An ISI total score ≥ 8 was classified as the insomnia group, while scores of 0–7 were categorized as the non-insomnia group (26). The ISI has been widely used as a validated measure of insomnia in studies of Chinese populations (27). The PHQ-9 comprises 9 self-reported items, each scored from 0 to 3, with a total score range of 0-27. A PHQ-9 score ≥ 10 indicated the presence of current depression, while scores of 0–9 indicated no depression (28). The GAD-7 contains 7 self-report items, each scored from 0 to 4, with a total score range of 0-21. A GAD-7 score ≥ 10 was classified as the presence of current anxiety, while scores of 0–9 were classified as no anxiety (29).

### 2.3 Statistical analyses

Data analysis was conducted using SPSS version 25.0. Missing data were handled by complete case analysis. Categorical data were expressed as frequencies and percentages, and group comparisons were conducted using the Chi-square test. Univariate analysis was first performed to explore potential factors affecting insomnia among urban and rural populations. Insomnia status (whether or not insomnia was present) was then used as the dependent variable, and variables with significant differences in univariate analysis were included as independent variables. Multivariate logistic regression was ultimately applied to explore the influencing factors of insomnia among urban and rural populations. Statistical significance was set at a two-tailed P-value of < 0.05.

## 3 Results

### 3.1 Comparison of basic characteristics between urban and rural residents

A total of 9,251 questionnaires were collected in this survey, and after excluding 207 invalid responses (including missing data), 9,044 valid questionnaires were included in the study, with a valid response rate of 97.76%. Among the respondents, there were 4,546 urban residents (50.27%) and 4,498 rural residents (49.73%).

There were no statistically significant differences between urban and rural residents in terms of napping habits, regular diet, or the prevalence of depression and anxiety (P > 0.05). Moreover, urban residents exhibited significantly higher proportions in several aspects compared to rural residents (P < 0.05): female gender, middle-aged and older populations, high educational attainment, unmarried status, widowed/divorced status, middle-to-high monthly household incomes, and professional technical occupations or retired status. In contrast, rural residents demonstrated significantly greater proportions in the following areas: male gender, younger age groups, low educational levels, married status, and lower monthly household income (P < 0.05). Furthermore, urban residents displayed healthier lifestyle habits, including a significantly higher frequency of physical exercise and lower proportions of current smoking and drinking compared to rural residents (P < 0.05). Finally, findings indicated that the prevalence of chronic diseases and insomnia was significantly higher among urban residents than rural residents (P < 0.05). Specifically, the overall insomnia prevalence among Foshan residents was 18.37%. Urban residents had a significantly higher insomnia prevalence (19.73%) than rural residents (16.99%), as illustrated in **Table 1**.

**Table 1:** Comparison of basic characteristics between urban and rural residents.

Variables	Total (N=9044)	Urban (N=4546)	Rural (N=4498)	χ2	P
	[n (%)]	[n (%)]	[n (%)]		
Gender					
Male	4359 (48.20)	2092 (46.02)	2267 (50.40)	17.386	<0.001^*^
Female	4685 (51.80)	2454 (53.98)	2231 (49.60)		
Age					
18–45 years	5567 (61.55)	2698 (59.35)	2869 (63.78)	18.832	<0.001^*^
45–65 years	2816 (31.14)	1499 (32.97)	1317 (29.28)		
≥65 years	661 (7.31)	349 (7.68)	312 (6.94)		
Educational years					
≤6 years	788 (8.71)	305 (6.71)	483 (10.74)	107.070	<0.001^*^
6–12 years	4114 (45.49)	1936 (42.59)	2178 (48.42)		
≥12 years	4142 (45.80)	2305 (50.70)	1837 (40.84)		
Marital status					
Married	6989 (77.28)	3436 (75.58)	3553 (78.99)	16.465	<0.001^*^
Unmarried	1566 (17.32)	834 (18.35)	732 (16.27)		
Widowed/Divorced	489 (5.41)	276 (6.07)	213 (4.74)		
Monthly household income					
≤3500 ¥	3705 (40.97)	1698 (37.35)	2007 (44.62)	101.643	<0.001^*^
3500-9000 ¥	4728 (52.28)	2438 (53.63)	2290 (50.91)		
≥9000 ¥	611 (6.76)	410 (9.02)	201 (4.47)		
Occupation					
Worker/Farmer	2088 (23.09)	770 (16.94)	1318 (29.3)	315.876	<0.001^*^
Public officer/Technician/Military	1774 (19.62)	1000 (22.00)	774 (17.21)		
Business/Service/Logistics support	960 (10.61)	562 (12.36)	398 (8.85)		
Retired personnel	1126 (12.45)	738 (16.23)	388 (8.63)		
Others	3096 (34.23)	1476 (32.47)	1620 (36.02)		
Smoking status					
No	7160 (79.17)	3695 (81.28)	3465 (77.03)	31.176	<0.001^*^
Used to smoke, now quit	309 (3.42)	160 (3.52)	149 (3.31)		
Current regular smoker	1575 (17.41)	691 (15.20)	884 (19.65)		
Alcohol consumption					
No	7960 (88.01)	4054 (89.18)	3906 (86.84)	12.122	0.002^*^
Used to drink, now quit	310 (3.43)	136 (2.99)	174 (3.87)		
Current regular drinker	774 (8.56)	356 (7.83)	418 (9.29)		
Tea-drinking habit					
No	5147 (56.91)	2509 (55.19)	2638 (58.65)	11.018	0.001^*^
Yes	3897 (43.09)	2037 (44.81)	1860 (41.35)		
Napping habit					
No	1524 (16.85)	733 (16.12)	791 (17.59)	3.447	0.063
Yes	7520 (83.15)	3813 (83.88)	3707 (82.41)		
Diet regularly					
No	286 (3.16)	147 (3.23)	139 (3.09)	0.152	0.697
Yes	8758 (96.84)	4546 (100.00)	4359 (96.91)		
Exercise frequency					
Hardly	4364 (48.25)	2056 (45.23)	2308 (51.31)	39.526	<0.001^*^
1–2 times/week	1739 (19.23)	885 (19.47)	854 (18.99)		
3–5 times/week	904 (10.00)	490 (10.78)	414 (9.20)		
almost daily	2037 (22.52)	1115 (24.53)	922 (20.50)		
Chronic diseases					
No	6410 (70.88)	3134 (68.94)	3276 (72.83)	16.597	<0.001^*^
Yes	2634 (29.12)	1412 (31.06)	1222 (27.17)		
Depression					
No	8582 (94.89)	4310 (94.81)	4272 (94.98)	0.130	0.718
Yes	462 (5.11)	236 (5.19)	226 (5.02)		
Anxiety					
No	8787 (97.16)	4409 (96.99)	4378 (97.33)	0.979	0.322
Yes	257 (2.84)	137 (3.01)	120 (2.67)		
Insomnia					
No	7383 (81.63)	3649 (80.27)	3734 (83.01)	11.374	0.001^*^
Yes	1661 (18.37)	897 (19.73)	764 (16.99)		

*P < 0.05.

### 3.2 Univariate analysis of insomnia among urban and rural residents

Univariate analysis results of insomnia prevalence among urban and rural residents are illustrated in **Table 2**. For urban residents, differences in insomnia occurrence by education level, marital status, occupation, smoking status, drinking status, dietary regularity, exercise frequency, chronic diseases, depression symptoms, and anxiety symptoms were all statistically significant (P < 0.05). For rural residents, statistically significant differences in insomnia occurrence were observed across variables including gender, age, education level, marital status, dietary habits, exercise frequency, chronic illnesses, depression, and anxiety (P < 0.05).

**Table 2:** Univariate analysis of factors Influencing insomnia among urban and rural residents.

Variables	Urban (N=4546)				Rural (N=4498)			
	Total (N)	Insomnia [n (%)]	χ2	P	Total (N)	Insomnia [n (%)]	χ2	P
Gender			0.339	0.560			11.156	0.001^*^
Male	2092	405 (19.36)			2267	343 (15.13)		
Female	2454	492 (20.05)			2231	421 (18.87)		
Age			4.151	0.125			14.121	0.001^*^
18–45 years	2698	548 (20.31)			2869	519 (18.09)		
45–65 years	1499	272 (18.15)			1317	182 (13.82)		
≥65 years	349	77 (22.06)			312	63 (20.19)		
Educational years			8.360	0.015^*^			13.754	0.001^*^
≤6 years	305	72 (23.61)			483	82 (16.98)		
6–12 years	1936	347 (17.92)			2178	326 (14.97)		
≥12 years	2305	478 (20.74)			1837	356 (19.38)		
Marital status			34.816	<0.001^*^			16.523	<0.001^*^
Married	3436	611 (17.78)			3553	563 (15.85)		
Unmarried	834	209 (25.06)			732	151 (20.63)		
Widowed/Divorced	276	77 (27.90)			213	50 (23.47)		
Monthly household income			3.252	0.197			5.283	0.071
≤3500 ¥	1698	319 (18.79)			2007	339 (16.89)		
3500-9000 ¥	2438	485 (19.89)			2290	379 (16.55)		
≥9000 ¥	410	93 (22.68)			201	46 (22.89)		
Occupation			13.315	0.010^*^			1.447	0.836
Worker/Farmer	770	125 (16.23)			1318	211 (16.01)		
Public officer/Technician/Military	1000	231 (23.10)			774	135 (17.44)		
Business/Service/Logistics support	562	112 (19.93)			398	68 (17.09)		
Retired personnel	738	144 (19.51)			388	65 (16.75)		
Others	1476	285 (19.31)			1620	285 (17.59)		
Smoking status			7.623	0.022^*^			1.544	0.462
No	3695	713 (19.30)			3465	599 (17.29)		
Used to smoke, now quit	160	45 (28.13)			149	27 (18.12)		
Current regular smoker	691	139 (20.12)			884	138 (15.61)		
Alcohol consumption			15.783	<0.001^*^			3.501	0.174
No	4054	769 (18.97)			3906	648 (16.59)		
Used to drink, now quit	136	41 (30.15)			174	36 (20.69)		
Current regular drinker	356	87 (24.44)			418	80 (19.14)		
Tea-drinking habit			0.005	0.944			0.641	0.423
No	2509	496 (19.77)			2638	458 (17.36)		
Yes	2037	401 (19.69)			1860	306 (16.45)		
Napping habit			1.390	0.238			0.588	0.443
No	733	133 (18.14)			791	127 (16.06)		
Yes	3813	764 (20.04)			3707	637 (17.18)		
Diet regularly			51.294	<0.001^*^			51.878	<0.001^*^
No	147	63 (42.86)			139	55 (39.57)		
Yes	4546	834 (18.35)			4359	709 (16.27)		
Exercise frequency			15.380	<0.001^*^			19.693	<0.001^*^
Hardly	2056	440 (21.40)			2308	439 (19.02)		
1–2 times/week	885	167 (18.87)			854	141 (16.51)		
3–5 times/week	490	110 (22.45)			414	68 (16.43)		
almost daily	1115	180 (16.14)			922	116 (12.58)		
Chronic diseases			101.979	<0.001^*^			88.589	<0.001^*^
No	3134	493 (15.73)			3276	451 (13.77)		
Yes	1412	404 (28.61)			1222	313 (25.61)		
Depression			350.399	<0.001^*^			449.310	<0.001^*^
No	4310	739 (17.15)			4272	609 (14.26)		
Yes	236	158 (66.95)			226	155 (68.58)		
Anxiety			288.862	<0.001^*^			277.622	<0.001^*^
No	4409	792 (17.96)			4378	676 (15.44)		
Yes	137	105 (76.64)			120	88 (73.33)		

*P < 0.05.

### 3.3 Logistic regression analysis of factors influencing insomnia among Foshan residents

#### 3.3.1 Logistic regression analysis of factors influencing insomnia among urban residents

Using urban residents’ insomnia status in Foshan as the dependent variable (No = 0, Yes = 1), 10 factors with statistically significant differences in univariate analysis (P < 0.1), including education level, marital status, occupation, smoking status, alcohol consumption, dietary regularity, exercise frequency, chronic diseases, depression, and anxiety, were included as independent variables in a multivariate logistic regression model. The results showed that unmarried and widowed/divorced status, professional technical occupations (e.g., public officer, technician, military personnel), chronic illness, depression, and anxiety were risk factors for insomnia. In contrast, having 6–12 years of education, regular eating habits, and daily exercise were identified as protective factors, as shown in **Table 3**.

**Table 3:** Multivariate logistic regression analysis of factors influencing insomnia among urban residents.

Variables	B	S.E.	Wald	P	OR	95%CI
Educational years (Ref: ≤ 6 years)						
6–12 years	-0.383	0.159	5.813	0.016^*^	0.682	0.499-0.931
≥12 years	-0.269	0.172	2.439	0.118	0.764	0.546-1.071
Marital status (Ref: Married)						
Unmarried	0.289	0.107	7.251	0.007^*^	1.335	1.082-1.647
Widowed/Divorced	0.467	0.154	9.147	0.002^*^	1.595	1.179-2.159
Occupation (Ref: Worker/Farmer)						
Public officer/Technician/Military	0.396	0.141	7.862	0.005^*^	1.490	1.127-1.961
Business/Service/Logistics support	0.189	0.157	1.453	0.228	1.210	0.888-1.643
Retired personnel	0.152	0.150	1.027	0.311	1.160	0.868-1.562
Others	0.176	0.129	1.860	0.173	1.190	0.926-1.536
Smoking status (Ref: No)						
Used to smoke, now quit	0.234	0.208	1.259	0.262	1.263	0.840-1.901
Current regular smoker	-0.117	0.125	0.871	0.351	0.890	0.696-1.137
Alcohol consumption (Ref: No)						
Used to drink, now quit	0.358	0.226	2.502	0.114	1.430	0.918-2.227
Current regular drinker	0.196	0.155	1.597	0.206	1.217	0.897-1.650
Diet regularly (Ref: No)						
Yes	-0.670	0.205	10.662	0.001^*^	0.512	0.342-0.765
Exercise frequency (Ref: Hardly)						
1–2 times/week	-0.049	0.109	0.207	0.649	0.952	0.769-1.177
3–5 times/week	0.146	0.131	1.246	0.264	1.157	0.895-1.496
almost daily	-0.309	0.113	7.521	0.006^*^	0.734	0.588-0.915
Chronic diseases (Ref: No)						
Yes	0.764	0.088	75.502	<0.001^*^	2.147	1.807-2.551
Depression (Ref: No)						
Yes	1.596	0.165	93.851	<0.001^*^	4.933	3.572-6.812
Anxiety (Ref: No)						
Yes	1.828	0.232	62.347	<0.001^*^	6.222	3.953-9.796

*P < 0.05. B, Regression Coefficient; S.E., Standard Error; OR, Odds Ratio; CI, Confidence Interval.

#### 3.3.2 Logistic regression analysis of factors influencing insomnia among rural residents

Using rural residents’ insomnia status in Foshan as the dependent variable (No = 0, Yes = 1), 10 factors with statistically significant differences in univariate analysis (P < 0.1), including gender, age, education level, marital status, monthly household income, dietary regularity, exercise frequency, chronic diseases, depression, and anxiety, were included as independent variables in a multivariate logistic regression model. The results showed that female gender, having ≥ 12 years of education, high household income, chronic illness, and symptoms of depression or anxiety were identified as risk factors for insomnia. Conversely, regular eating habits and daily exercise were protective factors, as presented in **Table 4**.

**Table 4:** Multivariate logistic regression analysis of factors influencing insomnia among rural residents.

Variables	B	S.E.	Wald	P	OR	95%CI
Gender (Ref: Male)						
Female	0.399	0.090	19.723	<0.001^*^	1.491	1.250-1.779
Age (Ref: 18–45 years)						
45–65 years	-0.079	0.126	0.397	0.528	0.924	0.722-1.182
≥65 years	0.309	0.219	2.000	0.157	1.362	0.888-2.091
Educational years (Ref: ≤6 years)						
6–12 years	0.156	0.174	0.799	0.371	1.169	0.830-1.645
≥12 years	0.451	0.198	5.221	0.022^*^	1.570	1.066-2.313
Marital status (Ref: Married)						
Unmarried	0.234	0.123	3.615	0.057	1.263	0.993-1.608
Widowed/Divorced	0.359	0.188	3.652	0.056	1.432	0.991-2.070
Monthly household income (Ref: ≤3500¥)						
3500-9000 ¥	0.005	0.096	0.003	0.957	1.005	0.832-1.214
≥9000 ¥	0.476	0.198	5.756	0.016^*^	1.609	1.091-2.374
Diet regularly (Ref: No)						
Yes	-0.784	0.210	13.887	<0.001^*^	0.457	0.302-0.690
Exercise frequency (Ref: Hardly)						
1–2 times/week	0.010	0.115	0.007	0.932	1.010	0.806-1.265
3–5 times/week	-0.046	0.153	0.090	0.764	0.955	0.707-1.290
almost daily	-0.332	0.128	6.714	0.010^*^	0.718	0.558-0.922
Chronic diseases (Ref: No)						
Yes	0.904	0.099	83.965	<0.001^*^	2.469	2.035-2.996
Depression (Ref: No)						
Yes	2.088	0.167	157.225	<0.001^*^	8.069	5.822-11.183
Anxiety (Ref: No)						
Yes	1.754	0.242	52.641	<0.001^*^	5.780	3.598-9.285

*P < 0.05. B, Regression Coefficient; S.E., Standard Error; OR, Odds Ratio; CI, Confidence Interval.

## 4 Discussion

This study provides a comprehensive analysis of urban-rural disparities in the prevalence and contributing factors of insomnia. To our knowledge, it represents one of the largest sample sizes of its kind conducted in China to date. We found that the insomnia prevalence among adult residents in Foshan was 18.37%, which is lower than the 24.8% prevalence rate reported for Guangdong Province (30), indicating that Foshan’s adult insomnia rate is relatively low compared to the provincial average. Meanwhile, consistent with previous research findings, this study found that the insomnia rate among urban residents was higher than that among rural residents (23, 24, 30). This disparity may stem from significant differences between urban and rural areas in sociodemographic characteristics, lifestyle, economic development levels, and healthcare service quality.

Further logistic regression analysis indicated that chronic diseases, depression, and anxiety are shared risk factors for insomnia among urban and rural populations. The close association between chronic diseases and insomnia has been well-established in earlier research (14, 30). Chronic disease patients often suffer from prolonged physical pain or discomfort, which directly leads to difficulty falling asleep and reduced sleep quality (31). Additionally, psychological stress caused by health concerns, side effects of long-term medication use, and biological rhythm disruptions due to endocrine or immune disorders may indirectly worsen insomnia symptoms (32). Meanwhile, depression and anxiety share a notable bidirectional relationship with insomnia (33–36). On the one hand, insomnia frequently manifests as a clinical symptom of depression and anxiety disorders (16, 37), with significantly higher insomnia prevalence observed in individuals with these conditions compared to the general population (38). Moreover, the severity of depression or anxiety is often positively correlated with the severity of insomnia symptoms (39). Conversely, insomnia symptoms typically improve as depression or anxiety treatment progresses (34). On the other hand, insomnia itself has been demonstrated to trigger or intensify depressive and anxious emotions (6, 9, 40). A 12-months cohort study showed that insomnia significantly increases the risk of persistent anxiety and depressive symptoms (41). Mason et al. (34) also verified that depression and anxiety levels are markedly higher among individuals with insomnia than those without the condition. Furthermore, interventions targeting insomnia effectively improve patients’ depression and anxiety symptoms (42, 43). This bidirectional interaction is likely mediated by abnormal neurotransmission, genetic polymorphisms, HPA dysregulation, structural and functional brain impairments (35, 44).

Moreover, the study identified that unmarried, widowed/divorced status and professional technical occupations (e.g., public officer, technician, military personnel) are specific risk factors for insomnia in urban populations. Unmarried and widowed/divorced individuals often experience higher levels of social isolation and lack of emotional support, particularly in the absence of close social relationships, which can significantly impair sleep quality (45, 46). Professional technical occupations are more susceptible to insomnia because of the high-intensity work stress, strong sense of responsibility, and irregular schedules that prevent adequate relaxation. These findings suggest that the fast-paced urban lifestyle and high-pressure environment impose substantial psychological burdens on residents, consequently disturbing their sleep.

In contrast, the risk factors for insomnia among rural residents are more closely linked to gender roles, educational attainment, and household income. In rural settings, women typically shoulder greater responsibilities for household tasks and caregiving, including child-rearing and elder care. The psychological stress from such traditional gender role expectations can greatly impact their sleep quality. Additionally, rural residents with higher educational attainment (≥ 12 years) may be more likely to feel the gap between societal expectations and actual circumstances, such as limited career development or a lack of job opportunities matching their education level (47). Such cognitive imbalance can result in psychological stress and anxiety, ultimately impairing sleep quality (48). This study also found that higher household monthly income is a risk factor for insomnia among rural residents, consistent with previous studies (49, 50). Previous study found that among relatively poor individuals, a 10% increase in relative income could result in an average reduction of 6–8 minutes in sleep duration (49). This may be because higher-income rural households often bear greater work burdens and social responsibilities, such as contracting land, running businesses, or participating in competitive labor markets. These economic activities may result in overwork and an accelerated pace of life, even reducing sleep duration. In summary, insomnia risks vary significantly across individuals with differing social and cultural backgrounds. Urban residents’ insomnia risk mainly stems from high-pressure work environments and social isolation, whereas rural residents are more affected by gender roles, educational opportunities, and income disparities. These differential factors highlight the need to account for individual social contexts and lifestyles when designing insomnia prevention and intervention strategies, ensuring more tailored and effective solutions. For urban areas, mental health support programs could be prioritized, especially for individuals in high-stress occupations or those experiencing social isolation. In contrast, rural areas may benefit from socioeconomic support to address unique stressors related to gender roles, education, and family income.

In line with prior studies, this research found that maintaining regular dietary and exercise routines serves as a shared protective factor against insomnia among urban and rural populations (51–54). Prior research indicates that regular diet and exercise can regulate gut microbiota, thereby modulating the release of neurotransmitters and microbial metabolites to enhance sleep quality (55). Additionally, aerobic exercise has been shown to significantly improve sleep quality by stimulating melatonin production, modulating autonomic nervous system activity, and regulating hormone secretion (56). Adhering to regular dietary and exercise routines can also mitigate psychological stress, thus lowering the likelihood of insomnia stemming from anxiety, depression, or emotional fluctuations (57, 58). Notably, among urban residents, 6–12 years of education is considered a protective factor against insomnia. This might be attributed to their stronger workplace adaptability and relatively balanced life pressures. Conversely, those with lower educational attainment may experience significant financial and survival pressures due to unstable income, whereas highly educated individuals might grapple with psychological strain stemming from elevated expectations (47, 48). In this study, higher education levels (≥ 12 years) were even found to be a risk factor for insomnia among rural residents. This finding highlights the multifaceted influence of education on insomnia risk, particularly in the differing social contexts of urban and rural settings. These results emphasize both the critical role of consistent healthy lifestyle practices in reducing insomnia and the broader implications of education and social environments on mental health.

This study has several limitations. First, the cross-sectional design employed in this study limits the ability to capture the dynamic nature of sleep conditions over time and to establish causal relationships between influencing factors. Future studies should prioritize large-scale, high-quality longitudinal designs to gain a deeper understanding of the mechanisms underlying insomnia and its temporal dynamics. Second, data on insomnia, anxiety, and depression were collected via self-reported questionnaires, which are susceptible to subjective factors like recall bias and social desirability bias, potentially compromising the accuracy of the findings. Third, the study did not differentiate between insomnia subtypes, such as difficulty initiating sleep (DIS), difficulty maintaining sleep (DMS), and early morning awakening (EMA), which may limit a detailed understanding of insomnia patterns (59). Fourth, the data in this study were collected from Foshan City, which may limit the generalizability of the findings to other regions or populations in China. Lastly, although this study explored several factors potentially influencing insomnia, it did not account for other relevant variables, such as work stress, electronic device usage, and environmental conditions. Seasonal variations and long-term lifestyle changes, which could also impact sleep quality, were not considered. Future research should comprehensively assess the combined impact of these factors on sleep.

## 5 Conclusions

In conclusion, this study demonstrates significant urban-rural differences in the prevalence of insomnia among Foshan residents, with urban residents exhibiting higher insomnia rates than rural residents. The factors influencing insomnia display both shared characteristics and distinct differences between urban and rural populations. The study offers a scientific basis for managing insomnia among Foshan’s urban and rural populations and underscores the intricate mechanisms of insomnia across diverse social environments. For both urban and rural residents, attention should be given to the insomnia issues of those with chronic diseases, anxiety, and depressive disorders, providing them with psychological support and sleep interventions. Moreover, promoting regular healthy diets and physical exercise as universal methods can significantly improve residents’ sleep quality. For urban residents, special attention should be given to individuals experiencing marital difficulties (e.g., unmarried, widowed, or divorced) and professional technical occupations. In rural settings, it is essential to focus on highly educated individuals and address sleep health inequities arising from gender disparities and income gaps. These findings may also have broader implications for other rapidly urbanizing regions globally, where similar urban-rural disparities in mental health and lifestyle factors could contribute to varying insomnia prevalence. The insights from this study could inform targeted public health strategies in other regions, helping to mitigate insomnia and its related impacts on mental health.

## Data Availability

The raw data supporting the conclusions of this article will be made available by the authors, without undue reservation.

## References

[1] Morin CM Jarrin DC. Epidemiology of insomnia: prevalence, course, risk factors, and public health burden. Sleep Med Clin. (2022) 17:173–91. doi: 10.1016/j.jsmc.2022.03.003 35659072

[2] AlRasheed MM Fekih-Romdhane F Jahrami H Pires GN Saif Z Alenezi AF. The prevalence and severity of insomnia symptoms during COVID-19: A global systematic review and individual participant data meta-analysis. Sleep Med. (2022) 100:7–23. doi: 10.1016/j.sleep.2022.06.020 36030616 PMC9359588

[3] Chen Y Kartsonaki C Clarke R Guo Y Yu C Bian Z. Characteristics and correlates of sleep duration, daytime napping, snoring and insomnia symptoms among 0.5 million Chinese men and women. Sleep Med. (2018) 44:67–75. doi: 10.1016/j.sleep.2017.11.1131 29530372 PMC5869948

[4] Baril AA Beiser AS Sanchez E Mysliwiec V Redline S Gottlieb DJ. Insomnia symptom severity and cognitive performance: Moderating role of APOE genotype. Alzheimers Dement. (2022) 18:408–21. doi: 10.1002/alz.12405 PMC8802306 34310026

[5] Léger D Massuel MA Metlaine A. Professional correlates of insomnia. Sleep. (2006) 29:171–8. doi: 10.1093/sleep/29.2.171 16494084

[6] Wu TT Zou YL Xu KD Jiang XR Zhou MM Zhang SB. Insomnia and multiple health outcomes: umbrella review of meta-analyses of prospective cohort studies. Public Health. (2023) 215:66–74. doi: 10.1016/j.puhe.2022.11.021 36645961

[7] Zhang X Sun Y Ye S Huang Q Zheng R Li Z. Associations between insomnia and cardiovascular diseases: a meta-review and meta-analysis of observational and Mendelian randomization studies. J Clin Sleep Med. (2024) 20:1975–84. doi: 10.5664/jcsm.11326 PMC11609828 39167428

[8] Hu S Lan T Wang Y Ren L. Individual insomnia symptom and increased hazard risk of cardiocerebral vascular diseases: A meta-analysis. Front Psychiatry. (2021) 12:654719. doi: 10.3389/fpsyt.2021.654719 34054612 PMC8160242

[9] Hertenstein E Benz F Schneider CL Baglioni C. Insomnia-A risk factor for mental disorders. J Sleep Res. (2023) 32:e13930. doi: 10.1111/jsr.13930 37211915

[10] Salo P Vahtera J Ferrie JE Akbaraly T Goldberg M Zins M. Trajectories of sleep complaints from early midlife to old age: longitudinal modeling study. Sleep. (2012) 35:1559–68. doi: 10.5665/sleep.2210 PMC3466803 23115405

[11] Pajėdienė E Urbonavičiūtė V Ramanauskaitė V Strazdauskas L Stefani A. Sex differences in insomnia and circadian rhythm disorders: A systematic review. Medicina (Kaunas). (2024) 60:474. doi: 10.3390/medicina60030474 38541200 PMC10971860

[12] Sosso FAE Matos E Papadopoulos D. Social disparities in sleep health of African populations: A systematic review and meta-analysis of observational studies. Sleep Health. (2023) 9:828–45. doi: 10.1016/j.sleh.2023.08.021 37880077

[13] Etindele Sosso FA Torres Silva F Queiroz Rodrigues R Carvalho MM Zoukal S Zarate GC. Prevalence of sleep disturbances in Latin American populations and its association with their socioeconomic status-A systematic review and a meta-analysis. J Clin Med. (2023) 12:7508. doi: 10.3390/jcm12247508 38137577 PMC10743597

[14] Muhammad T Das M Jana A Lee S. Sex differences in the associations between chronic diseases and insomnia symptoms among older adults in India. Nat Sci Sleep. (2024) 16:1339–53. doi: 10.2147/nss.S456025 PMC11401520 39282468

[15] Mookerjee N Schmalbach N Antinori G Thampi S Windle-Puente D Gilligan A. Comorbidities and risk factors associated with insomnia in the elderly population. J Prim Care Community Health. (2023) 14:21501319231168721. doi: 10.1177/21501319231168721 37070688 PMC10123921

[16] Nutt D Wilson S Paterson L. Sleep disorders as core symptoms of depression. Dialogues Clin Neurosci. (2008) 10:329–36. doi: 10.31887/DCNS.2008.10.3/dnutt PMC3181883 18979946

[17] Hu N Wang C Liao Y Dai Q Cao S. Smoking and incidence of insomnia: a systematic review and meta-analysis of cohort studies. Public Health. (2021) 198:324–31. doi: 10.1016/j.puhe.2021.07.012 34507139

[18] Hu N Ma Y He J Zhu L Cao S. Alcohol consumption and incidence of sleep disorder: A systematic review and meta-analysis of cohort studies. Drug Alcohol Depend. (2020) 217:108259. doi: 10.1016/j.drugalcdep.2020.108259 32927195

[19] Boyle JT Nielson SA Perlis ML Dzierzewski JM. Move your feet and sleep: A longitudinal dynamic analysis of self-reported exercise, sedentary behavior, and insomnia symptoms. Sleep Health. (2024) 10:321–6. doi: 10.1016/j.sleh.2024.02.005 PMC11162937 38548566

[20] Xie Y Liu S Chen XJ Yu HH Yang Y Wang W. Effects of exercise on sleep quality and insomnia in adults: A systematic review and meta-analysis of randomized controlled trials. Front Psychiatry. (2021) 12:664499. doi: 10.3389/fpsyt.2021.664499 34163383 PMC8215288

[21] Zhong BL Li HJ Xu YM Jiang XF. Clinical insomnia among elderly primary care attenders in Wuhan, China: A multicenter cross-sectional epidemiological study. Front Public Health. (2022) 10:1026034. doi: 10.3389/fpubh.2022.1026034 36339226 PMC9634545

[22] Tang J Liao Y Kelly BC Xie L Xiang YT Qi C. Gender and regional differences in sleep quality and insomnia: A general population-based study in Hunan province of China. Sci Rep. (2017) 7:43690. doi: 10.1038/srep43690 28262807 PMC5337959

[23] Liu L Xue P Li SX Zhang J Zhou J Zhang W. Urban-rural disparities in mental health problems related to COVID-19 in China. Gen Hosp Psychiatry. (2021) 69:119–20. doi: 10.1016/j.genhosppsych.2020.07.011 PMC7395944 32868097

[24] Zheng W Luo XN Li HY Ke XY Dai Q Zhang CJ. Regional differences in the risk of insomnia symptoms among patients from general hospital outpatient clinics. Neuropsychiatr Dis Treat. (2018) 14:3307–15. doi: 10.2147/ndt.S184216 PMC6280893 30555236

[25] Xiang YT Ma X Cai ZJ Li SR Xiang YQ Guo HL. The prevalence of insomnia, its sociodemographic and clinical correlates, and treatment in rural and urban regions of Beijing, China: a general population-based survey. Sleep. (2008) 31:1655–62. doi: 10.1093/sleep/31.12.1655 PMC2603488 19090321

[26] Wong ML Lau KNT Espie CA Luik AI Kyle SD Lau EYY. Psychometric properties of the Sleep Condition Indicator and Insomnia Severity Index in the evaluation of insomnia disorder. Sleep Med. (2017) 33:76–81. doi: 10.1016/j.sleep.2016.05.019 28449911

[27] Zhang QQ Li L Zhong BL. Prevalence of insomnia symptoms in older Chinese adults during the COVID-19 pandemic: A meta-analysis. Front Med (Lausanne). (2021) 8:779914. doi: 10.3389/fmed.2021.779914 34869501 PMC8634335

[28] Costantini L Pasquarella C Odone A Colucci ME Costanza A Serafini G. Screening for depression in primary care with Patient Health Questionnaire-9 (PHQ-9): A systematic review. J Affect Disord. (2021) 279:473–83. doi: 10.1016/j.jad.2020.09.131 33126078

[29] Sapra A Bhandari P Sharma S Chanpura T Lopp L. Using generalized anxiety disorder-2 (GAD-2) and GAD-7 in a primary care setting. Cureus. (2020) 12:e8224. doi: 10.7759/cureus.8224 32582485 PMC7306644

[30] Shan W Peng X Tan W Zhou Z Xie H Wang S. Prevalence of insomnia and associations with depression, anxiety among adults in Guangdong, China: A large-scale cross-sectional study. Sleep Med. (2024) 115:39–47. doi: 10.1016/j.sleep.2024.01.023 38330694

[31] Ohayon MM. Relationship between chronic painful physical condition and insomnia. J Psychiatr Res. (2005) 39:151–9. doi: 10.1016/j.jpsychires.2004.07.001 15589563

[32] Ditmer M Gabryelska A Turkiewicz S Białasiewicz P Małecka-Wojciesko E Sochal M. Sleep problems in chronic inflammatory diseases: prevalence, treatment, and new perspectives: A narrative review. J Clin Med. (2021) 11:67. doi: 10.3390/jcm11010067 35011807 PMC8745687

[33] Cai L Bao Y Fu X Cao H Baranova A Zhang X. Causal links between major depressive disorder and insomnia: A Mendelian randomization study. Gene. (2021) 768:145271. doi: 10.1016/j.gene.2020.145271 33122081

[34] Mason EC Harvey AG. Insomnia before and after treatment for anxiety and depression. J Affect Disord. (2014) 168:415–21. doi: 10.1016/j.jad.2014.07.020 25108278

[35] Riemann D Krone LB Wulff K Nissen C. Sleep, insomnia, and depression. Neuropsychopharmacology. (2020) 45:74–89. doi: 10.1038/s41386-019-0411-y 31071719 PMC6879516

[36] Johnson EO Roth T Breslau N. The association of insomnia with anxiety disorders and depression: exploration of the direction of risk. J Psychiatr Res. (2006) 40:700–8. doi: 10.1016/j.jpsychires.2006.07.008 16978649

[37] Cutler AJ. The role of insomnia in depression and anxiety: its impact on functioning, treatment, and outcomes. J Clin Psychiatry. (2016) 77:e1010. doi: 10.4088/JCP.14076tx3c 27561147

[38] Oh CM Kim HY Na HK Cho KH Chu MK. The effect of anxiety and depression on sleep quality of individuals with high risk for insomnia: A population-based study. Front Neurol. (2019) 10:849. doi: 10.3389/fneur.2019.00849 31456736 PMC6700255

[39] Jansson-Fröjmark M Norell-Clarke A Linton SJ. The role of emotion dysregulation in insomnia: Longitudinal findings from a large community sample. Br J Health Psychol. (2016) 21:93–113. doi: 10.1111/bjhp.12147 26347204

[40] Mendelsohn AK Daffre C Oliver K Seo J Lasko N Pace-Schott E. 765 Anxiety and sleep in Generalized Anxiety Disorder with and without Insomnia Disorder. Sleep. (2021) 44:A298–8. doi: 10.1093/sleep/zsab072.762

[41] Meaklim H Saunders WJ Byrne ML Junge MF Varma P Finck WA. Insomnia is a key risk factor for persistent anxiety and depressive symptoms: A 12-month longitudinal cohort study during the COVID-19 pandemic. J Affect Disord. (2023) 322:52–62. doi: 10.1016/j.jad.2022.11.021 36372131

[42] Staines AC Broomfield N Pass L Orchard F Bridges J. Do non-pharmacological sleep interventions affect anxiety symptoms? A meta-analysis. J Sleep Res. (2022) 31:e13451. doi: 10.1111/jsr.13451 34331373

[43] Boland EM Goldschmied JR Gehrman PR. Does insomnia treatment prevent depression? Sleep. (2023) 46:zsad104. doi: 10.1093/sleep/zsad104 37029781 PMC10262035

[44] Benca RM Peterson MJ. Insomnia and depression. Sleep Med. (2008) 9 Suppl 1:S3–9. doi: 10.1016/s1389-9457(08)70010-8 18929317

[45] Kawata Y Maeda M Sato T Maruyama K Wada H Ikeda A. Association between marital status and insomnia-related symptoms: findings from a population-based survey in Japan. Eur J Public Health. (2020) 30:144–9. doi: 10.1093/eurpub/ckz119 31280305

[46] Lindström M Rosvall M. Marital status, social capital, economic stress, and mental health: a population-based study. Soc Sci J. (2012) 49:339–42. doi: 10.1016/j.soscij.2012.03.004 22925881

[47] Schoger LI. Coping with work-related stressors: does education reduce work-related stress? J Public Health. (2023) 33:1123-34. doi: 10.1007/s10389-023-02070-5

[48] Koopman C Wanat SF Whitsell S Westrup D Matano RA. Relationships of alcohol use, stress, avoidance coping, and other factors with mental health in a highly educated workforce. Am J Health Promot. (2003) 17:259–68. doi: 10.4278/0890-1171-17.4.259 12640782

[49] Akay A Martinsson P Ralsmark H. Relative concerns and sleep behavior. Econ Hum Biol. (2019) 33:1–14. doi: 10.1016/j.ehb.2018.12.002 30591424

[50] Seo WH Kwon JH Eun SH Kim G Han K Choi BM. Effect of socio-economic status on sleep. J Paediatr Child Health. (2017) 53:592–7. doi: 10.1111/jpc.13485 28573803

[51] Eun H-B Baek S-S Eun H-B Baek S-S. Effect of exercise on sleep in the middle-aged and older adult: A systematic review and meta-analysis of randomized controlled trials. Exercise Sci. (2023) 32:21–32. doi: 10.15857/ksep.2023.32.1.21

[52] Riedel A Benz F Deibert P Barsch F Frase L Johann AF. The effect of physical exercise interventions on insomnia: A systematic review and meta-analysis. Sleep Med Rev. (2024) 76:101948. doi: 10.1016/j.smrv.2024.101948 38749363

[53] Zheng YB Huang YT Gong YM Li MZ Zeng N Wu SL. Association of lifestyle with sleep health in general population in China: a cross-sectional study. Transl Psychiatry. (2024) 14:320. doi: 10.1038/s41398-024-03002-x 39098892 PMC11298538

[54] Arab A Karimi E Garaulet M Scheer F. Dietary patterns and insomnia symptoms: A systematic review and meta-analysis. Sleep Med Rev. (2024) 75:101936. doi: 10.1016/j.smrv.2024.101936 38714136 PMC11179690

[55] Liu L Zhu JW Wu JL Li MZ Lu ML Yu Y. Insomnia and intestinal microbiota: a narrative review. Sleep Breath. (2024) 29:10. doi: 10.1007/s11325-024-03206-x 39589434

[56] Okechukwu CE Masala D D’Ettorre G La Torre G. Moderate-intensity aerobic exercise as an adjunct intervention to improve sleep quality among rotating shift nurses. Clin Ter. (2022) 173:184–6. doi: 10.7417/ct.2022.2414 35385043

[57] Shen Q Wang S Liu Y Wang Z Bai C Zhang T. The chain mediating effect of psychological inflexibility and stress between physical exercise and adolescent insomnia. Sci Rep. (2024) 14:24348. doi: 10.1038/s41598-024-75919-8 39420219 PMC11486977

[58] Brupbacher G Gerger H Zander-Schellenberg T Straus D Porschke H Gerber M. The effects of exercise on sleep in unipolar depression: A systematic review and network meta-analysis. Sleep Med Rev. (2021) 59:101452. doi: 10.1016/j.smrv.2021.101452 33667885

[59] Xu YM Li C Zhu R Zhong BL. Prevalence and correlates of insomnia symptoms in older Chinese adults during the COVID-19 outbreak: A classification tree analysis. J Geriatr Psychiatry Neurol. (2022) 35:223–8. doi: 10.1177/08919887221078561 PMC8899830 35245996

